# Stepped wedge randomised controlled trials: systematic review of studies published between 2010 and 2014

**DOI:** 10.1186/s13063-015-0839-2

**Published:** 2015-08-17

**Authors:** Emma Beard, James J. Lewis, Andrew Copas, Calum Davey, David Osrin, Gianluca Baio, Jennifer A. Thompson, Katherine L. Fielding, Rumana Z. Omar, Sam Ononge, James Hargreaves, Audrey Prost

**Affiliations:** Department of Clinical, Educational and Health Psychology, University College London, 1-19 Torrington Place, London, WC1E 7HB UK; Department of Epidemiology and Public Health, University College London, 1-19 Torrington Place, London, WC1E 7HB UK; MRC Tropical Epidemiology Group, Department of Infectious Disease Epidemiology, London School of Hygiene and Tropical Medicine, Keppel Street, London, WC1E 7HT UK; MRC Clinical Trials Unit at University College London, 175 Tottenham Court Road, London, W1T 7NU UK; Department of Social and Environmental Health Research, London School of Hygiene and Tropical Medicine, Keppel Street, London, WC1E 7HT UK; Institute for Global Health, University College London, 30 Guilford Street, London, WC1N 1EH UK; Department of Statistical Science, University College London, 1-19 Torrington Place, London, WC1E 7HB UK; Department of Infectious Disease Epidemiology, London School of Hygiene and Tropical Medicine, Keppel Street, London, WC1E 7HT UK; Department of Obstetrics and Gynaecology, Makerere University College of Health Sciences, P.O. Box 7072, Kampala, Uganda

**Keywords:** Stepped wedge trials, Systematic review, Methodology, Public health

## Abstract

**Background:**

In a stepped wedge, cluster randomised trial, clusters receive the intervention at different time points, and the order in which they received it is randomised. Previous systematic reviews of stepped wedge trials have documented a steady rise in their use between 1987 and 2010, which was attributed to the design’s perceived logistical and analytical advantages. However, the interventions included in these systematic reviews were often poorly reported and did not adequately describe the analysis and/or methodology used. Since 2010, a number of additional stepped wedge trials have been published. This article aims to update previous systematic reviews, and consider what interventions were tested and the rationale given for using a stepped wedge design.

**Methods:**

We searched PubMed, PsychINFO, the Cumulative Index to Nursing and Allied Health Literature (CINAHL), the Web of Science, the Cochrane Library and the Current Controlled Trials Register for articles published between January 2010 and May 2014. We considered stepped wedge randomised controlled trials in all fields of research. We independently extracted data from retrieved articles and reviewed them. Interventions were then coded using the functions specified by the Behaviour Change Wheel, and for behaviour change techniques using a validated taxonomy.

**Results:**

Our review identified 37 stepped wedge trials, reported in 10 articles presenting trial results, one conference abstract, 21 protocol or study design articles and five trial registrations. These were mostly conducted in developed countries (n = 30), and within healthcare organisations (n = 28). A total of 33 of the interventions were educationally based, with the most commonly used behaviour change techniques being ‘instruction on how to perform a behaviour’ (n = 32) and ‘persuasive source’ (n = 25). Authors gave a wide range of reasons for the use of the stepped wedge trial design, including ethical considerations, logistical, financial and methodological. The adequacy of reporting varied across studies: many did not provide sufficient detail regarding the methodology or calculation of the required sample size.

**Conclusions:**

The popularity of stepped wedge trials has increased since 2010, predominantly in high-income countries. However, there is a need for further guidance on their reporting and analysis.

**Electronic supplementary material:**

The online version of this article (doi:10.1186/s13063-015-0839-2) contains supplementary material, which is available to authorized users.

## Background

Methods for designing, analysing and reporting cluster randomised trials are now well established [1, 2]. A potential alternative to randomising clusters to a simple treatment or control condition is to randomly allocate the time at which clusters receive an intervention. This is termed a ‘stepped wedge’ trial design. Consequently, all clusters have received the intervention by the end of the trial. Other terms for this trial design found in the literature include experimentally staged introduction, delayed intervention and phased implementation trials. A stepped wedge trial based on randomising the time at which individuals, rather than clusters, receive the intervention is possible, but uncommon in the literature [3].

Two systematic reviews have been published on stepped wedge randomised controlled trials (SWTs). The first was conducted by Brown and Lilford [4] in March 2006 and identified 12 protocols or articles. They included both randomised and non-randomised studies, and those with allocations at individual and cluster level. However, they limited the review to the health sector. They concluded that there were regularities in the motivation for adopting the stepped wedge design, but that the methodological descriptions of studies, including the sample size calculations and analytical methods, were not always complete. Sample size calculations were reported in only five out of 12 studies, and there was considerable variation in the analytical methods applied.

Mdege et al. [5] updated Brown and Lilford’s review and expanded the search to include non-healthcare trials, but focussed only on randomised studies with cluster allocations. They retrieved 25 articles up to January 2010. Common reasons given for choosing a stepped wedge design were perceived methodological and logistical benefits, as well as improved social acceptability based on the premise that every cluster would eventually receive the intervention. Mdege et al. also identified problems with the clarity of reporting and analysis.

These systematic reviews concluded that the stepped wedge design was gaining in popularity, but that the studies were often poorly reported. The use of the stepped wedge design in randomised controlled trials is likely to have increased after the publication of articles by Hussey and Hughes [6] and Moulton et al. [7] in 2007, which described sample size calculations and analytical methods for SWTs involving dichotomous and/or continuous outcomes, and survival data. Poor reporting likely results from the lack of standardised Consolidated Standards for Reporting Trials (CONSORT) guidelines [8, 9].

There have been additional publications on the reporting, analysis and/or sample size calculations for SWTs [10–12] since Mdege et al.’s review [5]. At the same time, controversy around the use of this design has increased in the literature. Some authors have raised objections to the reasons given for conducting SWTs. For example, Kotz et al. [13] argued that the ability to roll out an intervention to all clusters for ethical reasons is not an inherent property of SWTs, and should not form the basis of choice over a traditional parallel cluster randomised controlled trials: it is possible to have a wait-list control group in a cluster randomised controlled trial, or to implement the intervention in the control group if beneficial effects are found. Other concerns raised by researchers include the often longer duration of SWTs, the possibility of increased drop-out rates due to repeated measurements and a concern that an intervention may be implemented in all clusters, which has not yet been proven to be effective. There is also an active debate in the literature about the conditions under which SWTs may have greater or less statistical power than parallel trials [9, 14, 15]. Mdege et al. have subsequently agreed with many of these arguments, however, they have also pointed out that although they may hold for the evaluation of healthcare treatments, they do not generally hold for policy-type trials, for which the alternative is often no randomised trial at all [16]. These issues are discussed in more detail in the other papers which make up this special issue of trials [17–19].

As part of this collection of articles on SWTs, we updated previous systematic reviews to:

1. Determine how many protocols and articles have subsequently been recorded,
2. Describe the areas of study and countries in which the design was most commonly used,
3. Identify the types of intervention which have been evaluated using SWTs,
4. Examine the stated reasons for conducting SWTs,
5. Identify the main design features, and
6. Describe the methods used to calculate sample sizes and to analyse data.

The current paper focuses on objectives one to three (Additional file 1 sections: 1.1-1.3, 2.1-2.3, 2.6-2.8, 3.1, 3.2, 3.5-3.9, 3.12-3.14, 5.1-5.3). Objectives four to six are considered in more detail in the other articles in this issue of *Trials*.

## Methods

### Literature search

We searched the following sources: PubMed, PsycINFO, the Cumulative Index to Nursing and Allied Health Literature (CINAHL), the Web of Science, the Cochrane Library and the Current Controlled Trials Register. The search was conducted on 14 May 2014, and was limited to studies published or registered since 1 January 2010 and written in English. The search terms were any of the following in the abstract: ‘stepped wedge’, ‘step wedge’, ‘experimentally staged introduction’, ‘delayed intervention’ or ‘one directional cross over design’. All articles, conference abstracts, protocols and trial registrations of original randomised research studies that used or planned to use a stepped wedge design, from any field of research, were eligible. We excluded studies retrospectively analysed as a stepped wedge design when the study was not originally designed as a stepped wedge. Where original articles for studies included in the Mdege review as protocols had been published, the published articles were considered for inclusion. We also reviewed methodological and design articles on SWTs published since Mdege et al. [5] in order to understand current methodological debates. Some of these articles were identified through the formal literature search detailed above, and others by checking the reference lists of identified articles. These are reviewed in the other publications of this special issue of *Trials* [17–19].

### Review of studies

One author (AP) reviewed the titles and abstracts of all identified research articles, conference abstracts, protocols and trial registrations to decide on eligibility for full review. Another author (EB) then re-ran the search to double check that all eligible papers had been identified between 1 January 2010 and 14 May 2014. Pairs of authors then reviewed the full texts of selected articles. Studies subsequently identified as non-randomised or not a SWT, regardless of how they were described by study authors, were then removed. Any additional studies known to the authors of this article that met the eligibility criteria above were also included.

### Data extraction and analysis

Pairs of authors reviewed the full texts of articles screened by AP and used a standardised data extraction form to extract key information on each study (see Additional file 1). Relevant sections of these forms were then collated for this article by two authors (EB and JL). Additional, sections were collated by authors of the other papers in this special issue of *Trials* [17, 19, 18]. For conference abstracts or trial registrations, a number of these sections were not relevant and were coded as ‘not applicable’. Discrepancies between pairs of completed forms were resolved through discussions between EB and co-authors.

In order to characterise the types of interventions tested through SWTs, we categorised all interventions using the functions described by the Behaviour Change Wheel (BCW) framework [20]. Although many frameworks are available to categorise interventions (for example, MINDSPACE [21]), these have been criticised for their lack of comprehensibility and their conceptual incoherence [20]. The BCW stipulates nine types of intervention functions, which can be applied to various policy categories including regulation, fiscal measures, guidelines, environmental and social planning, communication and marketing, legislation and service provision. These nine functions are as follows: 1) education (increasing knowledge or understanding, for example, providing information to promote healthy eating), 2) persuasion (using communication to produce feelings that stimulate action, for example, using imagery to motivate increases in physical activity), 3) incentivisation (creating expectation of a reward, for example, using prize draws to increase medication adherence), 4) training (imparting skills, for example, training to increase safe cycling), 5) restriction (using rules to reduce the opportunity to engage in the behaviour of interest, for example, prohibiting the sale of solvents to those under 18-years-old), 6) environmental restructuring (changing the physical or social context, for example, providing free at-home gym equipment), 7) modelling (providing an example for people to imitate, for example, using television drama scenes to promote safe sex), 8) enablement (reducing barriers to an individual’s capability or opportunity, for example, medication for cognitive deficits) and 9) coercion (creating expectation of punishment or increasing cost, for example, raising the cost of cigarettes to reduce consumption). EB coded all interventions using these nine functions. A subset of papers was also coded by a researcher familiar with the BCW, until 90 % agreement was obtained. Any discrepancies were resolved through consensus discussions. For interventions using the education function, we specified whether this was for the client or healthcare professional.

EB then used a taxonomy of 93 Behaviour Change Techniques (BCT Taxonomy v1) [22], to describe the components of each intervention. Guidelines from Michie et al. [22, 23] were followed, including only coding BCTs when there was unequivocal evidence of their inclusion in a given intervention. The taxonomy includes a standard definition of, and detailed coding instructions for each BCT, including examples of instances in which each BCT should or should not be coded.

## Results

### Study selection

**Fig. 1:**
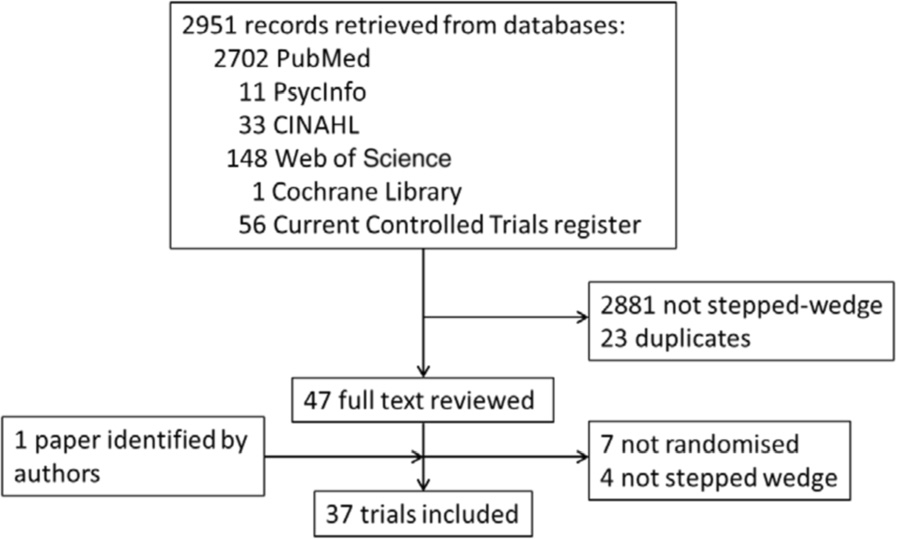
Flowchart describing the selection of studies included in the systematic review

Figure 1 describes the selection of studies included in this systematic review. Of the 2,948 records retrieved from the database search, we reviewed 47 full texts, and 36 studies were eligible for this review. In addition, the authors of this paper identified one more paper not found in the database search (as it is an SWT, but also refers to ‘stepped expansion’ in the abstract). Four of the published papers had previously been included as published protocols in the Mdege et al. review [5].

**Fig. 2:**
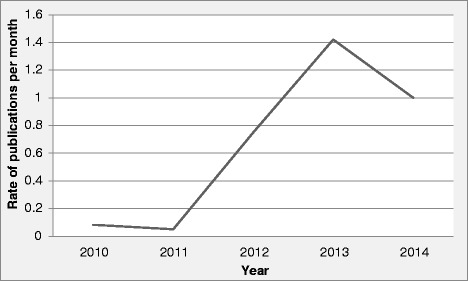
Rate of publications per month between 2010 and 2014

These 37 studies consisted of 10 articles presenting trial results, one conference abstract, 21 protocol or design articles and five trial registrations (Table 1) [24–62]. It is clear from Fig. 2 that the rate of publications on SWTs has increased between 2010 and 2014.

**Table 1:** Characteristics of included studies which adopted a stepped wedge randomised controlled trial design

First author	Study start date (publication date)	Study duration	Country	Intervention	Primary outcome	Cluster definition	Why did investigators choose stepped wedge trial design?
Presentation of trial results - research articles							
Bacchieri *et al*. [24]	2006 (2010)	20 months	Brazil	Education intervention to prevent traffic accidents among cyclists	Traffic accidents and near accidents	40 sectors within 5 neighbourhoods	Ethical - no equipoise; phased implementation - cannot implement in many clusters at same time
Bashour *et al*. [25]	2008 (2013)	10 months	Syria	Training resident doctors in interpersonal and communication skills	Women’s satisfaction with interpersonal and/or communication skills of doctors working in labour and delivery rooms	4 teaching public maternity hospitals	Ethical and practical
Durovni *et al*. [26]	2005 (2013)	42 months	Brazil	Implementation of widespread isoniazid preventive therapy for HIV-positive patients	Incidence of active tuberculosis	29 HIV clinics	Ethical - no equipoise (intervention recommended, but not implemented); phased implementation - cannot implement in many clusters at same time
Fuller *et al*. [27]	2006 (2012)	38 months	United Kingdom	Feedback intervention to improve hand hygiene compliance in UK healthcare workers	Hand hygiene compliance measured by observers blinded to the hospital allocation	16 hospitals	Ethical - no equipoise; phased implementation - cannot implement in many clusters at same time; prevent contamination and disappointment effects in control hospitals; clusters act as own controls so higher statistical power; extended duration allows assessment of sustainability
Gruber *et al*. [28]	2009 (2013)	15 months	Mexico	Ultraviolet-disinfection system designed to treat household drinking water.	Proportion of households with contaminated drinking water and 7-day prevalence of diarrhoea (co-primary)	24 rural communities	Phased implementation - cannot implement in many clusters at same time
Horner *et al*. [29]	2006 (2012)	28 months	United Kingdom	Staff training and education on the topic of infection prevention and effective hand hygiene	Prevalence of MRSA infection	65 care homes	Allow measurement of prevalence before the intervention, directly after the intervention and further follow-up in two of the three study groups; participating residents and staff in each group of homes acted as controls for each other
Mhurchu *et al*. [30]	2010 (2013)	11 months	New Zealand	Free daily before-school breakfast programme	The proportion of students achieving a school attendance of 95 % or higher	16 schools	None given
Kitson *et al*. and Schultz *et al*. [31, 32]	2011 (2013/2014)	12 months	Australia	A multifaceted intervention incorporating a malnutrition screening tool, nutritional supplements and red trays	Rate of change in body mass index over weekly periods from admission to discharge	25 hospital wards	Political - intervention is to be rolled out to all clusters eventually; ethical - no equipoise; phased implementation - cannot implement in many clusters at same time; improvements can be made to the intervention; temporal changes in effectiveness can be modelled; clusters act as own controls so higher statistical power
Roy *et al*. [33]	2009 (2013)	7 months	United Kingdom	Universal offer of testing without detailed pre-test discussion; training of clinic staff; and the provision of tailor-made information material for patients and healthcare workers	HIV test acceptance amongst those offered a test	24 tuberculosis clinics	Political - intervention to be rolled out to all clusters eventually
Stern *et al*. [34]	2010 (2014)	17 Months	Canada	Educating staff on the prevention and treatment of pressure ulcers; use of Enhanced Multi-Disciplinary Team (EMDT)	Rate of reduction in pressure ulcer surface area	12 long-term care facilities	Desire to have benefits of randomization; ethical - no equipoise; phased implementation - cannot implement in many clusters at same time
Conference abstracts							
Fearon *et al*. [35]	2013 (2013)	15 months	United Kingdom	Telephone hotline to link GPs directly with stroke patients’ specialists for: immediate discussion, treatment advice, prioritisation of investigations	Reduction in the time from referral to specialist stroke team input	72 GP practices	None given
Trial protocol/design articles							
Bennett *et al*. [36]	2013 (2013)	12 months	Australia	Accredited exercise physiologist coordinated program on physical function	Objective physical function measured using the 30-second sit to stand test.	15 haemodialysis clinics	Ethical - no equipoise; phased implementation - cannot implement in many clusters at same time
Bernabe-Ortiz *et al*. [37]	2012 (2014)	7 months	Peru	Population-level social marketing campaign to introduce a low-sodium, high potassium salt substitute	Blood pressure and use of salt	6 villages	Phased implementation - cannot implement in many clusters at same time
Brimblecombe *et al*. [38]	2012 (2013)	12 months	Australia	Price intervention: 20 % discount on food in store. Combined intervention: price discount and in-store nutrition education strategy	Per capita daily weight of combined fruit and vegetables purchased through the community store.	20 communities	Phased implementation - cannot implement in many clusters at same time
Dainty *et al*. [39]	2010 (2011)	24 months	Canada	Multi-faceted knowledge translation strategy designed to increase the utilisation rate of induced hypothermia in survivors of cardiac arrest	Proportion of survivors of cardiac arrest presenting to the emergency department that achieve the target temperature within six hours of ED arrival.	37 hospitals	Ethical - no equipoise (intervention recommended, but not implemented); phased implementation - cannot implement in many clusters at same time; temporal changes in effectiveness can be modelled
Dreischulte *et al*. [40]	2011 (2012)	96 months	United Kingdom	Data-Driven Quality Improvement in Primary Care (DQIP) with three components: education, informatics and financial incentive	Composite score of prescribing outcomes	40 GP practices	Phased implementation - cannot implement in many clusters at same time; prevents control clusters dropping out; higher statistical power
Gerritsen *et al*. [41]	2009 (2011)	24 months	Netherlands	Act In Case of Depression: multidisciplinary care program to improve the management of depression in nursing home residents	Frequency of depression and quality of life	32 somatic and dementia special care units	Higher statistical power; all clusters receive the intervention - expected to increase motivation of clusters to participate in the study
Gucciardi *et al*. [42]	2012 (2012)	24 months	Canada	Mobile diabetes education team (MDET) intervention to support primary care providers by offering a diabetes education team	Change in HbA1c (an index of diabetes control)	12 primary care sites	All participating physicians want the intervention; all clusters receive intervention - gives additional data on effectiveness
Keriel-Gascou *et al*. [43]	2012 (2013)	18 months	France	Interactive program that encouraged patients to report adverse drug events in primary care	Reporting of adverse drug events by antihypertensive-treated patients to their GPs	8 clusters of GP practices	Ethical - no equipoise; phased implementation - cannot implement in many clusters at same time; clusters act as own controls so higher statistical power; temporal changes in effectiveness can be modelled
Kjeken *et al*. [44]	2011 (2014)	10 months	Norway	New rehabilitation program PRAISE versus current rehabilitation program	Goal attainment and health-related quality of life	6 rehabilitation centres	Ethical - no equipoise; phased implementation - cannot implement in many clusters at same time
Marshall *et al*. [45]	2012 (2012)	18 months in first area and 12 months in second area	United Kingdom	Targeted case finding of patients at high risk of CVD versus opportunistic assessment	Number of high-risk patients started on at least one preventive treatment: an antihypertensive drug or a statin	32 GP practices in two areas	Phased implementation - cannot implement in many clusters at same time; evaluate effects of the case finding programme before and after implementation of intervention
Mouchoux *et al*. [46]	2011 (2011)	24 months	France	Multifaceted prevention program involving structured geriatric consultation, training sessions and practice analysing medical records	Post-operative delirium rate within 7 days after surgery	Surgical wards within 3 districts	Ethical - no equipoise; phased implementation - cannot implement in many clusters at same time; clusters act as own controls so higher statistical power; temporal changes in effectiveness can be modelled
Poldervaart *et al*. [47]	2013 (2013)	14 months	Netherlands	Use of the HEART score, a clinical prediction rule, to provide a simple, early and reliable predictor of cardiac risk	Occurrence of major adverse cardiac events	10 hospitals	Within-hospital comparison less confounded by case-mix differences than between hospitals; all hospitals receive intervention so provide data about implementation problems; gradual intervention implementation provides data about the process; all clusters receive the intervention - expected to increase motivation of clusters to participate in the study
Praveen *et al*. [48]	2013 (2013)	24 months	India	Clinical decision support system to assist health workers in making decisions to lower patients’ cardiovascular disease (CVD) risks	Difference in proportion of high risk individuals (with or without CVD) who are achieving optimal blood pressure levels (systolic <140 mmHg)	18 primary health care centres	Ensure all receive intervention
Rasmussen *et al*. [49]	2013 (2013)	15 months	Denmark	Multifaceted worksite intervention consisting of participatory economic, physical exercise and cognitive behavioural training for lower back pain.	Lower back pain is measured by days with and intensity of pain each month throughout the data collection period	21 clusters each consisting of one team, unless small teams in similar location	Phased implementation - cannot implement in many clusters at same time; all clusters receive the intervention - expected to increase motivation of clusters to participate in the study
Ratanawongsa *et al*. [50]	2009 (2012)	24 months	USA	Automated Telephone Support Management intervention to promote care manager efficiency	Physical and mental functional status and the number of days spent in bed due to illness	8 clusters of participants	Ethical - no equipoise; phased implementation - cannot implement in many clusters at same time; temporal changes in effectiveness can be modelled
Solomon *et al*. [51]	2011 (2012)	23 months	United Kingdom	Devon Active Villages intervention to improve participation in physical activity	Proportion of adults meeting recommended daily guidelines for the minimum level of physical activity	128 villages	Ethical - no equipoise; phased implementation - cannot implement in many clusters at same time
Stringer *et al*. [52]	2011 (2013)	48 months	Zambia	Implementation of clinical protocols, forms and systems by Quality Improvement (QI) teams; engagement of community health workers.	Community level all-cause mortality among those aged <60 years	42 primary healthcare facilities and their catchment areas	Ethical - no equipoise; phased implementation - cannot implement in many clusters at same time
Tirlea *et al*. [53]	2001 (2013)	9 months	Australia	Girls on the Go! Program aimed at increasing self-esteem and self-efficacy	The Rosenberg Self-Esteem Scale and the Eating Disorders Assessment	12 schools	None given
Turner *et al*. [54]	2011 (2011)	4 months	Australia	Brief tailored psychosocial intervention in cancer care with focused training and clinical supervision	Change in depression as measured by Hospital Anxiety and Depression Score	5 hospitals	Able to account for systematic differences between sites and times during the trial, and also for case-mix differences between patients
Van de Steeg *et al*. [55]	2011 (2012)	11 months	Netherlands	E-learning course about delirium aimed at nursing staff.	Percentage of patients screened for risk of delirium; sample size based on screening for delirium risk and the effect on knowledge	18 hospitals	Ethical - no equipoise; all clusters receive the intervention - expected to increase motivation of clusters to participate in the study; reduce contamination bias as each hospital acts as their own control; take into account the effect of time on outcomes measures
van Holland *et al*. [56]	2012 (2012)	32 months	Netherlands	Employees offered health surveillance programs to reduce sickness absence	Work ability, productivity and absenteeism	5 meat processing companies	Clusters act as own controls so higher statistical power and fewer confounding factors
Trial registrations							
Craine [57]	2012 (2011)	12 months	United Kingdom	Dried blood spit testing (DBS) for blood borne viral infections versus standard venepuncture-based testing	Change in blood-borne viral diagnostic testing rate in prisons with introduction of DBS	5 prisons	None given
Everingham [58]	2014 (2014)	21 months	United Kingdom	Quality improvement project to help staff deliver highest standard of care for emergency laparotomy patients	All-cause mortality at 90 days following surgery	90 hospitals	Control adoption bias; adjust for time-based changes in the background level; can offer to every site
Grande [59]	2014 (2012)	24 months	United Kingdom	Formalised, comprehensive procedure for carer support needs assessment, prioritisation and follow-up	Quality of life	6 hospice home care services	None given
Koeberlein-Neu [60]	2013 (2013)	17 months	Germany	An inter-professional medication therapy management	Change in the Medication Appropriateness Index Scores measured every three months	14 GP surgeries	Phased implementation - cannot implement in many clusters at same time
Williams [62]	2012 (2012)	24 months	United Kingdom	Physiotherapists trained in clinical reasoning skills via a clinical mentoring program	Function measured by The Patient Specific Functional Scale.	12 physiotherapists	None given

### Study characteristics

Randomisation was at the cluster level in 36 of 37 trials, with one being individually randomised in a two-step SWT [50]. There were 11 studies based in the United Kingdom, five in Australia, four in the Netherlands, three in Canada, two in Brazil, two in France and 10 based in other countries (Denmark, Germany, India, Mexico, New Zealand, Norway, Peru, Syria, United States and Zambia). A total of 28 studies were conducted within healthcare organisations (for example, general practices and hospitals), four were based in the community, two within schools, one within the prison-service, one in the workplace and one within supermarkets. The median length of the trials was 18 months (range 4 to 96 months) and the median number of clusters was 17 (range: 4 to 128 clusters; one trial did not state the number of clusters).

### Design

There were 13 trials that used a continuous recruitment short exposure design, which involves the continuous recruitment of participants as they become available and exposure to either the control or intervention condition (but usually not both) for a short period, typically common to all participants. Generally, measures were taken on a one-off basis for each participant.

There were 11 studies that used a closed cohort design, whereby all participants are identified at baseline and most or all experience both the control and the intervention. Generally, measures were either time-to-event or taken repeatedly at regular intervals. Another 11 studies adopted an open cohort design. These were most often community-based interventions. Many participants are exposed from the start of the study using this design, but some will leave the study, while others may join. Thus, although many experience both the control and the intervention, some will only experience one. Generally, these studies used cross-sectional surveys at the beginning and end of each step.

Two studies had different designs to the three types outlined above. For further details see the design paper in this collection [18]. Simple randomisation for the order of intervention roll-out was the most common randomisation method (n = 17), followed by stratified (n = 13) and restricted (n = 1) randomisation. Six trials did not report the randomisation method clearly.

### Intervention features

Numerous behaviours were targeted, including academic achievement and attendance, blood pressure, depression and hand hygiene (see Additional file 2). Using the functions of the BCW [15], thirty-three of the interventions included educational based components, four used persuasion, four used incentivisation, twenty used training (that is, imparted skills), eight used environmental restructuring, six used enablement, three used modelling and one used coercion. None were based on the function restriction. In 20 of the 33 trials with education components, the education was applied to healthcare professionals, in 12 trials it was applied to the client (for example, the patient) and in one trial the education component was applied to a mixture of people. The most commonly used BCTs were ‘instruction on how to perform a behaviour’ (n = 32), ‘persuasive source’ (*n* = 25), ‘adding objects to the environment’ (n = 14) and ‘restructuring the physical environment’ (n = 13).

### Reasons for use

A variety of reasons were given for adopting the SWT design, including ethical, logistical and methodological and/or analytic reasons (see Table 1). In 21 studies, authors felt that the logistical barriers to simultaneously implementing the intervention in many clusters were too high, and so opted for a stepped wedge design. In 16 studies, authors described a lack of equipoise for the intervention based on positive pilot study results or prior literature, and felt it would be unethical to deny the intervention to some groups. Another reason cited in eight trials was to avoid the ‘disappointment effects’ possible in a parallel trial, that is, to avoid some clusters dropping out of the study when randomised to the control arm. Since all clusters would receive the intervention at some point, this was thought by some to increase the motivation of health staff to participate. Two trials stated that the intervention was going to be rolled out to all clusters anyway.

Seven studies reported that the stepped wedge design would have higher statistical power, with five explicitly stating that this was because clusters would act as their own controls. Seven studies also reported that the ability to adjust for time trends in outcomes was an advantage. Six trials gave no explanation for adopting a stepped wedge design (including the one conference abstract and three trial registrations).

### Sample size calculations

Six of the studies did not report sample size calculations, or it was unclear whether they had been performed (including the one conference abstract and four trial registrations). Of those that did report sample size calculations, nine used a design effect for parallel or cluster randomised controlled trials. Those accounting for the stepped wedge design most commonly used the approach recommended by Hussey and Hughes [6]. One study used the design effect defined by Woertman et al. [10], and one used the method proposed by Moulton et al. [7]. Three trials used simulations to compute the sample size. The sample size calculations need to take the proposed analysis method into account, and these are complicated for stepped wedge trials; for further details see the sample size and analysis articles in this collection [17, 19].

## Discussion

The number of trials using stepped wedge designs appears to be increasing over time, with 37 new trials published or planned since the 25 identified in a previous review [5]. The trials identified in this latest review were mostly conducted in developed countries, in the health sector, and offered ethical, logistical and methodological reasons for adopting the design. Most interventions tested involved increasing knowledge through education or training, whether among staff providing a service or among clients, an effect which could be difficult to ‘remove’ in a two-way cross-over design (a design which randomises half the clusters to intervention and half to control for the first half of the trial, at which point they switch condition until the end of the trial [63]). However, the reporting of trial design and sample size calculations was generally poor.

There are some limitations to our review: we only included articles published in English and used only one trial register. We also did not search the reference lists of included studies. Another possible limitation is that we only focussed on studies published or registered since 1 January 2010. However, we wanted the review to reflect current practice and feel this choice is justified. In addition, we excluded studies (both implicitly through our search criteria and explicitly during full-text review) that did not use common terminology, and so may have missed some SWTs.

The rise in the number of studies adopting a stepped wedge design since 2010 could be a consequence of the publication of a handful of pivotal articles on sample size calculations and/or analysis of SWTs [6, 7, 10–12]. However, in line with the conclusions of Mdege et al. [5], some poor reporting remains. Clear descriptions were not always given for the rationale for using the stepped wedge design, the details of the design (including method of randomisation) or sample size calculations. This may partly be due to the lack of coherent recommendations for stepped wedge designs, with authors relying on those published previously for cluster randomised controlled trials. Although CONSORT type guidelines are being produced, these are not due for publication until 2017 [9]. Recommendations for this are beyond the scope of this article, but are discussed further in this collection [18, 19].

The reasons for using a stepped wedge design largely coincide with those reported previously: ethical, logistical and methodological [5, 4]. The potential impact of disappointment effects, whereby individuals not randomised to the treatment of choice fail to adhere or drop out, was given by several studies as a reason for choosing the SWT design (Table 1). However, some authors argue that this is not an inherent feature of SWTs, and that cluster randomised controlled trials can be extended to include a wait-list control group [13]. Thus the ethical argument that one should not withhold a potentially effective intervention from a group of individuals cannot form the sole justification for this trial design. It is possible that under certain circumstances, including the roll out of public health interventions, that a SWT would reduce required resources. One could easily envisage the situation of an intervention conducted by GPs, which would require one intervention trainer for an SWT (each GP is visited consecutively) and multiple for a cluster randomised controlled trial (each GP is trained concurrently). SWTs may also be suitable for optimising interventions, with the ability to modify content and delivery over time. However, the excess expense of this over factorial designs should be considered [64]. Finally, although it is possible under certain circumstances that the SWT design is optimal in terms of power, due partly to the within- and between-cluster data, this is not always the case [17, 14].

In line with the conclusions of Mdege et al. [5], the majority of SWTs we found were conducted in developed countries. However, there was an expansion beyond the earlier focus on nutrition and communicable diseases to a broad range of outcomes, including adverse drug event reporting, carer support and depression. The finding that the majority of interventions involved the functions of education and training is consistent with a previous review of 338 articles reporting on health-behaviour interventions [65]. The reliance on these likely reflects the adoption of common sense models of human behaviour during intervention development, that is, the long-held belief that improving knowledge and skills is sufficient to induce behaviour change in most circumstances [20]. There may also be a belief that education and training can do no harm, making them particularly appropriate to the stepped wedge design, with a lower requirement for equipoise than for a parallel design. However, if this is the case, we feel this may be simplistic, as training and education both come with opportunity costs for the time used to implement, as well as the potential to confuse or overburden participants. In addition, as explained in the third article of this collection (which is concerned with the logistics, ethics and politics of SWTs), we think that equipoise is still required for such trials [66].

All but one of the trials randomised the order of roll-out at the cluster level rather than the individual level. This may reflect the same logistical needs that lay behind the decision to opt for a stepped wedge rather than parallel design. SWTs used multiple designs, with important implications for analysis and sample size. These issues are discussed further in the relevant articles in this collection, but we note that there are several types of SWT and reporting the type of SWT used is important.

It is interesting that the first example of an SWT, the Gambia Hepatitis Intervention Study [63], was evaluating a new vaccine, yet none of the studies in this review were trialling a new medical intervention. Two studies investigated provision of isoniazid preventive therapy and HIV testing, but both of these were supported by current recommendations. Currently, questions related to equipoise, logistic benefits and increased social acceptability are leading to debates about the possible role of stepped wedge designs in the evaluation of new Ebola vaccines and treatments. In such circumstances, an important distinction may be drawn between vaccines and treatments, whereby vaccines may eventually be delivered to all participants, but treatments may come too late for those in the control condition. Clearly, the use of SWTs is increasing, and with this comes greater variety in trial contexts and designs, requiring further methodological work and guidance for researchers.

## Conclusions

This article aims to update previous systematic reviews on SWTs, consider what interventions were tested and the rationale given for using an SWT. The popularity of stepped wedge trials was found to have increased since 2010, predominantly in high-income countries. However, many were poorly reported and thus there is a need for further guidance on the conduction and reporting of SWTs.

## Additional files

Additional file 1:
**Data extraction form.** (DOCX 22 kb) Additional file 2:
**Further characteristics of studies that adopted a stepped wedge randomised controlled trial design included in the review.** (DOCX 34 kb)

## Abbreviations

CONSORT: Consolidated standards of reporting trials
SWT: Stepped wedge cluster randomised controlled trial
UK: United Kingdom
BCT: Behaviour change techniques
