# Supplementary material for: Stepped wedge randomised controlled trials: systematic review of studies published between 2010 and 2014
Source: Trials. 2015 Aug 17;16:353. doi: 10.1186/s13063-015-0839-2 (PMC4538902; doi:10.1186/s13063-015-0839-2)
Supplement: Additional file 1: — Data extraction form. (DOCX 22 kb) [file 13063_2015_839_MOESM1_ESM.docx]

**Additional file 1: Data extraction form**

| **Terminology** |  |
| --- | --- |
| **Intervention** | package of things that is randomized |
| **Cluster** | the unit of randomization |
| **Group** | the set of clusters that changes arm between steps |
| **Step** | the period when no groups change arm |
| **Step-change** | time when a group changes arm |

| **Reference** |  |
| --- | --- |
| **Study ID** |  |
| **First author** |  |
| **Year of study** |  |
| **Date form completed** |  |
| **Data extractor** |  |

| **Type of publication** | | Protocol  Presentation of findings  Other associated with trial  Registration |
| --- | --- | --- |
|  | 1. **General Information** |  |
| 1.1 | In which country is the study conducted? |  |
| 1.2 | How were the clusters defined? |  |
| 1.3 | What was the rationale for using the stepped-wedge design? |  |

|  | 1. **The Study** |  |
| --- | --- | --- |
| 2.1 | What interventions (including control conditions if appropriate) were compared? |  |
| 2.2 | What was the timing of the intervention delivery? | Immediate onset, continuous policy (e.g. a change to the treatment policy for patients)  Immediate onset, some delay in installation or set-up (e.g. cluster is initiated to receive new water and sanitation infrastructure but this takes time to construct)  Immediate onset, non-continuous (one-off) (e.g. mass de-worming of all eligible children in a cluster)  Repeat, discrete, interventions (e.g. six-monthly treatment of mosquito nets with insecticide)  Other: |
| 2.3 | What was the setting of implementation of intervention if different from cluster definition?  *e.g. households within clusters* |  |
| 2.4 | Was there potential for intervention effect to change over time? *e.g. when the intervention is a one-off training session, staff turn-over may mean that the intervention effect wanes over time* | What do authors say?  What do you – the reviewer – think?  None  Minor  Major  Details: |
| 2.5 | Was there potential for a time lag in intervention implementation? *e.g. set-up period, training*  (N.B . this may be considered a limitation so check in the discussion section)? | What do authors say?  What do you – the reviewer – think?  None  Minor  Major  Details: |
| 2.6 | What was the overall duration of the study? | Start date:  Stop date: |
| 2.7 | What were the primary outcomes? |  |
| 2.8 | What level were the primary outcomes measured at? *e.g. were the outcomes measured at the individual level, cluster level, or another unit such as the household?* |  |
| 2.9 | Was there potential for a time lag in outcome response to intervention implementation? *e.g. was there a long causal chain between the intervention and the outcomes, or was the outcome is based on very infrequent events?* | What do authors say?  What do you – the reviewer – think?  None  Minor  Major  Details: |
| 2.10 | Was there potential for the outcome response to the intervention to change over time, *e.g. the population becomes ‘immune’ over time, such as with a health promotion billboard campaign* | What do authors say? (if anything)  What do you – the reviewer – think?  None  Minor  Major  Details: |

|  | 1. **Methods** |  |
| --- | --- | --- |
| 3.1 | How did the authors do the randomization? | Simple random permutation  Stratified randomization  Restricted randomization  Other  Details: |
| 3.2 | Did the authors perform a sample size calculation prior to conducting the trial? | *YES/NO/UNCLEAR* |
| 3.3 | If ‘yes’ to 3.2: | Did the authors report an estimate for the within-cluster correlation in the outcome data (ignore if outcome measured at the cluster level)?  *YES/NO/UNCLEAR*  Was the estimated correlation derived from local or published data?  *YES/NO/UNCLEAR*  Did the authors account for the clustering in the sample size calculation?  *YES/NO/UNCLEAR*  Details:  Did the authors account for the stepped-wedge design in the sample size calculation?  *YES/NO/UNCLEAR*  Details:  Was the sample size calculation method appropriate for the model used in the analysis?  *YES/NO/UNCLEAR*  Details:  Did the authors account for missing data/loss to follow-up/refusal to participate in the sample size calculation?  *YES/NO/UNCLEAR*  Details: |
| 3.4 | What was the primary effect to be estimated? | Odds ratio  Risk ratio  Risk difference  Hazard ratio  Mean difference  Other  Details: |
| 3.5 | What was the total number of intervention arms? | 2 |
| 3.6 | What was the total number of clusters? | 20 |
| 3.7 | What was the total number of steps? | 5 |
| 3.8 | Number of clusters per group (the set of clusters that changes arm between steps) | 4 |
| 3.9 | What was the length of time between step changes? Please record if this was not consistent throughout the trial. | 2 months |
| 3.10 | Were outcome measurements collected from all clusters in a period before any receive the intervention? | *YES/NO/UNCLEAR*  If yes: were these measurements used in the analysis of intervention effect?  *YES/NO/UNCLEAR*  If yes: was this to  provide baseline measurements for adjustment  analyse changes in outcome over time  provide measurements to be included in the analysis like any other control condition measurements  other |
| 3.11 | Were outcome measurements collected from all clusters in a period after all clusters had started to receive the intervention? | *YES/NO/UNCLEAR*  If yes: were these measurements used in the analysis of intervention effect?  *YES/NO/UNCLEAR*  If yes: was this to  analyse changes in outcome over time  provide measurements to be included in the analysis like any other intervention condition measurements  other |
| 3.12 | How do data collection ‘units’ (individuals, households, clinics etc) participate in data collection? | *Closed cohort (i.e. recruitment at baseline)*  *Open cohort (i.e. participants are able to leave and to join the cohort as study progresses)*  *Continuous recruitment (i.e. participants are recruited as they become eligible)*  *Repeated closed cohorts (i.e. recruitment into a sequence of cohorts)*  *Other*  Details: |
| 3.13 | How are the primary outcome measurements obtained? | *Repeated measures of the same individuals (e.g. actively following up cohort members at regular intervals)*  *Repeated cross-sectional surveys of (mostly) different individuals (e.g. surveys of the ‘open cohort’ of people who live in a cluster)*  *One-off measurement of each individual (e.g. at the endlines of repeated closed cohorts)*  *Passive time to event (e.g. participants presenting with failure event)*  *Other*  Details:  If repeated measurements or cross-sectional surveys are used, how are these linked to the steps in the trial?  *Beginning of step*  *Beginning and end of step*  *Multiple times per step (number=____)*  *Other*  Details, including whether or not the *calendar time* of data collection varies between clusters:  Total number of measurements/surveys: |
| 3.14 | What was the assumed control outcome (e.g. at baseline) used in the sample size calculation? |  |
| 3.15 | For estimating the primary effect, was the comparison: within-step-between-clusters, between-step-within-cluster, both? |  |
| 3.16 | Was the analysis conducted at the individual or cluster-level? |  |
| 3.17 | What was the overall statistical model(s)? |  |
| 3.18 | What methods were used to address clustering? |  |
| 3.19 | What methods were used to address time trends? |  |
| 3.20 | What methods were used to address lag(s) (if appropriate)? |  |

|  | 1. **Results** |  |
| --- | --- | --- |
| 4.1 | What was the number of individuals in the entire study? |  |
| 4.2 | What was the completeness of follow-up/participation? | Number of clusters that dropped out of the study: _______  If closed-cohort, proportion of individuals enumerated at baseline(s) who dropped out/were lost to follow-up:_______  Proportion of data collection respondents (e.g. individuals) declining/unable to participate:_______  Any other details: |
| 4.3 | What is the mean number of data collection participants (e.g. individuals) per cluster? | *If one-off or repeated measures of the same individuals, mean number of individuals per cluster:______*  *If repeated cross-sectional surveys of different individuals, mean number of individuals per cluster:_______ and mean number of individuals per cluster in a survey:______*  *If passive time to event, mean number of events in each cluster:______*  *Other:* |
| 4.4 | Were baseline imbalances assessed? | *YES/NO/UNCLEAR*  Details: |
| 4.5 | Are potential confounders compared between before-intervention and after-intervention conditions? | *YES/NO/UNCLEAR*  Details: |
| 4.6 | Were there any significant baseline imbalances between the groups? | *YES/NO/UNCLEAR*  Details: |
| 4.7 | Is there lag in changes in the outcome relative to intervention implementation (see 2.5 and 2.9)? | *YES/NO/UNCLEAR*  Details: |
| 4.8 | Apart from lags, did the intervention effect change with time since initiation (see 2.4 and 2.10)? | *YES/NO/UNCLEAR*  Details: |

|  | 1. **Reporting** |  |
| --- | --- | --- |
| 5.1 | Is a CONSORT diagram present, and adapted to accommodate stepped-wedge design? |  |
| 5.2 | Is there a description, and/or figure, of SW design? |  |
| 5.3 | Are unadjusted cluster summaries reported? |  |
| 5.4 | Do the authors describe the trend in the outcome over time? |  |
| 5.5 | Did the authors report the actual extent of clustering in the data (e.g. ICC/K)? |  |
| 5.6 | Did the authors present baseline data to assess balance at baseline across groups or arm? |  |

|  | 1. **Risk of bias assessment** |  |
| --- | --- | --- |
| 6.1 | Was the intervention independent of other changes that might have influenced the outcomes? | *YES/NO/UNCLEAR*  Details: |
| 6.2 | Was the shape of the intervention effect pre-specified (i.e. lags)? | *YES/NO/UNCLEAR*  Details: |
| 6.3 | Were participants blinded to the allocation to intervention or control? | *YES/NO/UNCLEAR*  Details: |
| 6.4 | Were data collectors blinded to the allocation of clusters to intervention or control? | *YES/NO/UNCLEAR*  Details: |
| 6.5 | Were the researchers performing the analysis blinded to the allocation of clusters to intervention or control? | *YES/NO/UNCLEAR*  Details: |
