# Supplementary material for: Stepped wedge randomised controlled trials: systematic review of studies published between 2010 and 2014
Source: Trials. 2015 Aug 17;16:353. doi: 10.1186/s13063-015-0839-2 (PMC4538902; doi:10.1186/s13063-015-0839-2)
Supplement: Additional file 2: — Further characteristics of studies that adopted a stepped wedge randomised controlled trial design included in the review. (DOCX 34 kb) [file 13063_2015_839_MOESM2_ESM.docx]

**Additional file 2: Table S1:** Further characteristics of studies that adopted a stepped wedge RCT design included in the review

| **First author** | **Design typology** | **Randomisation method** | **Behaviours targeted** | **BCW components α** | **BCTs used β** | **Sample size calculation and method** |
| --- | --- | --- | --- | --- | --- | --- |
| **Presentation of trial results** | | | | | | |
| Bacchieri [19] | Closed cohort | Simple | Safe cycling | Education (Client)  Environmental restructuring  Enablement | Social support (unspecified)  Restructuring the physical environment  Adding objects to the environment  Instruction on how to perform a behaviour  Material incentive (behaviour)  Persuasive source  Pros and cons | Yes, unclear |
| Bashour [20] | Continuous recruitment short exposure | Simple | Patient satisfaction | Education (Health-care professional)  Enablement  Persuasion  Training | Self-monitoring of behaviour  Instruction on how to perform a behaviour  Persuasive source  Information about emotional consequences  Demonstration of behaviour  Social comparison | Yes, Hussey and Hughes |
| Durovni [21] | Open cohort | Restricted | Tuberculosis among patients with HIV | Education (Health-care professional) | Pharmacological support  Monitoring of behaviour by others without feedback  Instruction on how to perform a behaviour  Persuasive source | Yes, design effect from Moulton et al. |
| Fuller [22] | Open cohort | Simple | Hand Hygiene | Education (Health-care professional)  Persuasion  Coercion | Feedback on behaviour  Instruction on how to perform a behaviour  Non-specific reward  Goal setting (behaviour)  Action planning  Persuasive source | Yes, simulations |
| Gruber [23] | Closed cohort | Simple | Water contamination and diarrhoea | Environmental restructuring | Restructuring the physical environment  Adding objects to the environment | Yes, Hussey and Hughes |
| Horner [24] | Open cohort | Stratified | MRSA infection | Education (Health-care professional)  Training | Behavioural practice/rehearsal  Instruction on how to perform a behaviour  Persuasive source | Not given |
| Mhurchu [25] | Closed cohort | Simple | Attendance, hunger and academic achievement at school | Environmental restructuring | Restructuring the physical environment  Adding objects to the environment | Yes, Hussey and Hughes |
| Kitson/Schultz [26,27] | Continuous recruitment short exposure | Simple | Weight loss | Education (Client)  Training  Environmental restructuring | Restructuring the physical environment  Adding objects to the environment  Instruction on how to perform a behaviour  Persuasive source | Yes, Hussey and Hughes |
| Roy [28] | Continuous recruitment short exposure | Stratified | Offer of HIV testing | Education (Health-care professional)  Training | Instruction on how to perform a behaviour | Yes, design effect for parallel CRT |
| Stern [29] | Other | Simple | Pressure ulcer healing | Education (Health-care professional)  Training | Instruction on how to perform a behaviour  Persuasive source | Yes, simulations |
| **Conference abstracts** | | | | | | |
| Fearon [30] | Continuous recruitment short exposure | Simple | Assessment of minor stroke | Enablement | Adding objects to the environment | Not given |
| **Trial protocol/design papers** | | | | | | |
| Bennett [31] | Open cohort | Simple | Haemodialysis | Education (Health-care professional)  Training  Environmental restructuring | Monitoring outcome(s) of behaviour by others without feedback  Restructuring the physical environment  Adding objects to the environment  Instruction on how to perform a behaviour  Action planning  Persuasive source | Yes, design effect for parallel CRT |
| Bernabe-Ortiz [32] | Open cohort | Unclear | Dietary behaviour (reduced salt) | Education (Client)  Environmental restructuring  Enablement | Biofeedback  Restructuring the physical environment  Adding objects to the environment  Instruction on how to perform a behaviour  Persuasive source | Yes, Hussey and Hughes |
| Brimblecombe [33] | Closed cohort | Stratified | Dietary behaviour (food and alcohol) | Education (Client)  Incentivisation  Modelling | Instruction on how to perform a behaviour  Verbal persuasion about capability  Non-specific reward  Non-specific incentive  Persuasive source  Pros and cons  Framing/reframing  Demonstration of behaviour | Yes, simulations |
| Dainty [34] | Continuous recruitment short exposure | Stratified | Use of therapeutic hypothermia in post-cardiac arrest patients | Education (Health-care professional)  Training  Environmental restructuring | Social support (practical)  Feedback on behaviour  Self-monitoring of behaviour  Biofeedback  Restructuring the physical environment  Adding objects to the environment  Instruction on how to perform a behaviour | Post-hoc, Hussey and Hughes |
| Dreischulte [35] | Open cohort | Stratified | High-risk prescribing behaviour | Education (Health-care professional)  Incentivisation | Feedback on behaviour  Prompts/cues  Instruction on how to perform a behaviour  Material reward (behaviour)  Action planning  Persuasive source  Information about health consequences | Yes, Hussey and Hughes |
| Gerritsen [36] | Open cohort | Unclear | Depression | Education (Health-care professional)  Training | Social support (unspecified)  Pharmacological support  Monitoring of behaviour by others without feedback  Adding objects to the environment  Instruction on how to perform a behaviour  Action planning  Persuasive source | Yes, design effect for parallel CRT |
| Gucciardi [37] | Closed cohort | Stratified | Diabetes control | Education (Health-care professional) | Restructuring the social environment  Instruction on how to perform a behaviour  Persuasive source | Yes, Hussey and Hughes |
| Keriel-Gascou [38] | Continuous recruitment short exposure | Simple | Reporting of adverse drug events | Education (Client)  Training | Self-monitoring of behaviour  Instruction on how to perform a behaviour  Persuasive source  Information about health consequences | Yes, Hussey and Hughes |
| Kjeken [39] | Continuous recruitment short exposure | Simple | Rheumatic disorder rehabilitation | Education (Health-care professional)  Persuasion | Social support (practical)  Self-monitoring of behaviour  Prompts/cues  Instruction on how to perform a behaviour  Verbal persuasion about capability  Self-talk  Problem solving  Review behaviour goal(s)  Persuasive source | Yes, design effect for parallel CRT |
| Marshall [40] | Closed cohort | Stratified | Cardiovascular disease prevention | Education (Health-care professional) | Pharmacological support  Monitoring outcome(s) of behaviour by others without feedback  Adding objects to the environment  Instruction on how to perform a behaviour  Persuasive source  Pros and cons | Yes, design effect for parallel CRT |
| Mouchoux [41] | Continuous recruitment short exposure | Simple | Post-operative delirium | Education (Health-care professional)  Training  Environmental restructuring | Social support (unspecified)  Feedback on outcome(s) of behaviour  Monitoring of behaviour by others without feedback  Prompts/cues  Restructuring the physical environment  Adding objects to the environment  Instruction on how to perform a behaviour  Persuasive source  Information about health consequences | Yes, Hussey and Hughes |
| Poldervaart [42] | Continuous recruitment short exposure | Simple | Major adverse cardiac events | Education (Health-care professional)  Training | Monitoring of behaviour by others without feedback  Instruction on how to perform a behaviour | Yes, design effect for parallel CRT |
| Praveen [43] | Closed cohort | Stratified | Blood pressure | Education (Health-care professional)  Training | Restructuring the physical environment  Adding objects to the environment  Instruction on how to perform a behaviour  Persuasive source  Information about health consequences | Yes, Hussey and Hughes |
| Rasmussen [44] | Closed cohort | Stratified | Lower back pain | Education (Client)  Training | Feedback on behaviour  Restructuring the physical environment  Instruction on how to perform a behaviour  Action planning  Persuasive source  Pros and cons  Demonstration of behaviour | Yes, design effect from Woertman et al. |
| Ratanawongsa [45] | Closed cohort | Stratified | Diabetes self-management and real-time telephone support | Education (Client)  Training | Feedback on behaviour  Self-monitoring of behaviour  Instruction on how to perform a behaviour  Goal setting (behaviour)  Persuasive source | Yes, individually randomised and only two steps |
| Solomon [46] | Open cohort | Stratified | Physical activity | Education (Client)  Modelling  Enablement | Restructuring the physical environment  Adding objects to the environment  Instruction on how to perform a behaviour  Persuasive source | Yes, Hussey and Hughes |
| Stringer [47] | Open cohort | Stratified | Quality of clinical care and utilisation of care | Education (Health-care professional)  Training | Social support (practical)  Feedback on behaviour  Restructuring the physical environment  Instruction on how to perform a behaviour | Yes, design effect for parallel CRT |
| Tirlea [48] | Closed cohort | Stratified | Self-esteem | Education (Client)  Persuasion  Incentivisation  Modelling | Social support (practical)  Social support (emotional)  Instruction on how to perform a behaviour  Verbal persuasion about capability  Social reward  Goal setting (behaviour)  Action planning  Persuasive source  Information about health consequences  Information about emotional consequences  Demonstration of behaviour | Yes, design effect for parallel CRT |
| Turner [49] | Continuous recruitment short exposure | Simple | Psychosocial intervention for cancer patients | Education (Client)  Training | Social support (unspecified)  Monitoring of behaviour by others without feedback  Instruction on how to perform a behaviour  Action planning  Review behaviour goal(s)  Framing/reframing | Yes, design effect for parallel CRT |
| Van de Steeg [50] | Continuous recruitment short exposure | Simple | Delirium care | Education (Health-care professional)  Incentivisation  Training | Feedback on behaviour  Prompts/cues  Instruction on how to perform a behaviour  Material reward (behaviour)  Demonstration of behaviour | Yes, Hussey and Hughes |
| Van Holland [51] | Closed cohort | Simple | Work ability, productivity and sickness absence | Education (Client)  Training | Feedback on behaviour  Instruction on how to perform a behaviour  Persuasive source | Yes, Hussey and Hughes |
| **Trial registrations** | | | | | | |
| Craine [52] | Open cohort | Unclear | Dried blood spot testing | Education (Client) | Biofeedback | Not given |
| Everingham [53] | Continuous recruitment short exposure | Simple | Survival following laparotomy | Education (Health-care professional)  Training | Social support (unspecified)  Feedback on behaviour  Restructuring the physical environment  Adding objects to the environment  Instruction on how to perform a behaviour  Persuasive source  Framing/reframing  Social comparison | Yes, Hussey and Hughes |
| Grande [54] | Continuous recruitment short exposure | Unclear | Career support needs | Enablement | Social support (unspecified)  Monitoring of behaviour by others without feedback | Not given |
| Koeberlein-Neu [55] | Open cohort | Unclear | Medication therapy management | Education | Pharmacological support  Instruction on how to perform a behaviour | Not given |
| Williams [57] | Other | Unclear | Patient physical functioning following physiotherapy | Education (Health-care professional)  Training | Instruction on how to perform a behaviour  Persuasive source | Not given |

α Studies were coded using the functions specified by the Behaviour Change Wheel (BCW) [14]; β Behaviour Change Techniques (BCTs) were coded using a validated taxonomy [16]; CRT = cluster randomised trial
